# Mechanism of nodulation and nitrogen fixation in Caucasian clover (*Trifolium ambiguum* Bieb.) based on transcriptomics and proteomics analyses

**DOI:** 10.3389/fgene.2025.1600377

**Published:** 2025-07-24

**Authors:** Jiawei Liu, Xinyan Hao, Yue Guo, Mingjiu Wang, Fan Huang

**Affiliations:** ^1^ Institute of Plant Protection, Inner Mongolia Academy of Agricultural and Animal Husbandry Sciences, Hohhot, China; ^2^Institute of Grassland Research, Chinese Academy of Agricultural Science, Hohhot, China; ^3^ Research Department, Inner Mongolia University of Finance and Economics, Hohhot, China; ^4^College of Grassland Science, Inner Mongolia Agricultural University, Hohhot, China

**Keywords:** Caucasian clover, nodulation, nitrogen fixation, transcriptomics, proteomics

## Abstract

**Introduction:**

Caucasian clover (*Trifolium ambiguum* Bieb.), a perennial legume forage grass, exhibits strong adaptability and resistance to adverse conditions. Owing to its rhizome specificity, it cannot nodulate or fix nitrogen outside of its original location, which limits its promotion and cultivation. The phenomenon of spontaneous nodulation of Caucasian clover was observed for the first time in Inner Mongolia, and investigation of its nodulation and nitrogen fixation mechanisms assists in the subsequent promotion of Caucasian clover cultivation from a theoretical perspective.

**Methods:**

In this study, rhizobia extracted from Caucasian clover were inoculated into the field, and the nodulation part of the root system was investigated during the regreening stage of the second year using transcriptomics and proteomics techniques.

**Results and Discussion:**

The study identified 70,280 differentially expressed genes (DEGs) and 770 differentially expressed proteins (DEPs) in total, among which carbonic anhydrase, cyanate lyase, phenylalanine, caffeate/5-hydroxyferulate 3-O-methyltransferase, caffeoyl CoA 3-O methyltransferase, chalcone synthase, and chalcone isomerase may have been the main factors affecting the nodulation and nitrogen fixation of Caucasian clover. This study theoretically contributes to the future genetic validation and selection of Caucasian clover varieties with a strong ability to nodulate and fix nitrogen.

## 1 Introduction

Caucasian clover (*Trifolium ambiguum* Bieb.) is a long-rooted tiller legume forage native to Eastern Europe and the Caucasus region and exhibits excellent adaptability, cold resistance, and other natural endowments owing to the harsh local environment. Since the 1970s, Australia, New Zealand, the United States, Canada, and other countries have introduced and domesticated Caucasian clover and conducted numerous theoretical and applied studies, including studies on seed development characteristics (Hay et al., 2010), vegetative propagation characteristics (Baker, 2012), rhizobia and nitrogen fixation (Beauregard et al., 2003), and response to fertilizer (Sheaffer and Seguin, 2011).

Caucasian clover has the same nitrogen-fixing capacity and similar efficiency to that of white clover (Black et al., 2006). However, because of the selective specificity of Caucasian clover for rhizobacteria (Andrews et al., 2007; Miller et al., 2007), it can only be nodulated for nitrogen fixation with rhizobacteria from its places of origin, i.e., the Caucasus and parts of Eastern Europe, which affects its widespread cultivation. The Caucasian clover cultivar “*Trifolium ambiguum* Bieb. cv. Mengnong No. 1” (Inner Mongolia Agricultural University) can be tumor-fixed with rhizobacteria in Inner Mongolia, suggesting that Caucasian clover has great potential for nitrogen fixation via tumor fixation. Therefore, in this study, “*T. ambiguum* Bieb. cv. Mengnong No.1” was inoculated in the field, and its rhizosphere was analyzed using transcriptomics and a 4D label-free quantitative (LFQ) proteomic method with the aim of examining the gene and protein variation in the rhizosphere after rhizobial inoculation to guide the subsequent improvement of Caucasian clover. These findings are theoretically instrumental in the improvements in the nitrogen-fixation capacity of Caucasian clover.

Establishing a symbiotic nitrogen-fixing system involves a complex series of processes, including signal recognition and infection between the legume host crop and rhizobia, infestation line formation, rhizobial primordium formation, and root nodule maturation. Establishment of a symbiotic nitrogen-fixation relationship begins with the release of signaling substances and mutual recognition between the legumes and rhizobia. Legumes release metabolite flavonoid substances in the inter-root environment through the root system (Redmond et al., 1986), and when rhizobia successfully and specifically sense the flavonoid signal, those in the vicinity of the root system activate the expression of their own nodulation genes (*nod* genes) and release nodulation factors (*Nod* factors) around the crop roots (Caetano-Anollés et al., 1991; Spaink, 2000). *Nod* factors can induce nodule development in legume roots (Truchet et al., 1991). Thus, flavonoid compounds crucially impact the interactions between legumes and rhizobia. In *Lotus japonicus*, *Nod* factors may interact with the DFR-like protein, LjDFL1, to regulate flavonoid synthesis. Overexpression of DFL1 increases root invasion and promotes rhizobial infestation (Duan et al., 2019). In *Medicago truncatula*, downregulation of MtFNSII expression causes the depletion of flavonoids and interferes with rhizome formation. Flavonoids are important compounds in the early nodulation process, and phenylpropanoids also play an inducing role (Stafford, 1997).

Transcriptomic technology has emerged as a powerful tool for identifying key genes involved in symbiotic nitrogen fixation in legumes. By exploring the gene abundance in root nodules during the symbiotic interaction between legumes and rhizobia, this approach enables a comprehensive understanding of the molecular mechanisms underlying their mutualistic relationship. For instance, Hayashi et al. (2012) performed transcriptomic analysis on the nodulation zone of soybean roots inoculated with either wild-type rhizobia or a nodC mutant strain, identifying 2,915 genes that are specifically regulated by *Nod* factors produced by rhizobia. Similarly, Yuan et al. (2016) used RNA-seq to investigate differentially expressed genes (DEGs) in soybean roots interacting with different rhizobial strains. Their findings revealed that these genes primarily encode resistance proteins, nodulation factors, nodulins, immune defense proteins, and enzymes involved in flavonoid/flavone/flavonol biosynthesis and plant–pathogen interactions.

Proteomics has been extensively employed to study various aspects of legume crops, including growth and development, stress responses, and symbiotic interactions with rhizobia, thereby providing a solid foundation for elucidating the underlying molecular mechanisms. LV (2023) used proteomic techniques to demonstrate that the deletion of bjaR1 suppresses the expression of proteins associated with the nitrogen regulatory system. Chen et al. (2011) analyzed the impact of phosphorus (P) on the soybean nodule proteome, identifying 44 proteins that are responsive to P starvation—27 from the plant and 17 from the rhizobia. Their findings highlight the collaborative effort between soybean and rhizobia in adapting to P deficiency. Additionally, Salavati et al. (2012) compared the root proteomes of three soybean cultivars with varying nitrogen-fixing abilities (efficient: En-b0-1; inefficient: En1282; normal: Enrei) after rhizobial inoculation. They identified 56 differentially expressed proteins (DEPs) that are primarily involved in energy metabolism and signal transduction pathways.

Therefore, in this study, we inoculated *T*. *ambiguum* Bieb. ‘Mengnong No. 1’ with rhizobia under field conditions and employed transcriptomic and 4D label-free quantitative (LFQ) proteomic analyses to investigate changes in gene expression and protein abundance in the rhizosphere. Our aim was to characterize the molecular mechanisms underlying the symbiotic interaction between *T. ambiguum* and rhizobia, providing insights to guide the genetic improvement in this legume species. The findings of this study are expected to contribute theoretically to enhancing the nitrogen fixation efficiency in *T. ambiguum*.

## 2 Materials and methods

### 2.1 Experimental materials

The Caucasian clover variety “*Trifolium ambiguum Bieb. cv. Mengnong No.1”* bred by Inner Mongolia Agricultural University was used as the experimental material in this study.

The experimental strain was a rhizobacterium isolated from the root nodules of the variety and was identified as *Rhizobium leguminosarum* based on 16S rDNA analysis (Jiawei et al., 2023).

### 2.2 Experimental method

#### 2.2.1 Sampling method

Isolated rhizobia, after being inoculated into yeast mannitol agar (YMA) liquid medium, were cultured at at 180 r·min^−1^ and 28 C until the optical density value (OD_600_) exceeded 0.7, followed by centrifugation at 10,000 r·min^−1^ for 10 min. The resultant supernatant was resuspended in sterile water to achieve an optical density (OD_600_) of 0.7, which was then prepared for subsequent use (Duan et al., 2018).

The experiment consisted of two treatments: inoculation with rhizobia (NN) and a control group (CK). For the NN treatment, the prepared rhizobial suspension was inoculated into the root system of Caucasian clover (planted in July 2020) in August 2020. No rhizobial inoculation was applied to the CK treatment. Caucasian clover was sampled during its second year of re-greening.

#### 2.2.2 Determination of plant nutritional indices

The aboveground parts of plants were excised at the soil surface, first treated at 105 °C for 30 min, and then dried at 65° C to a constant weight. Nutritional indices, including neutral detergent fiber (NDF), acid detergent fiber (ADF), and crude protein (CP), were determined, with specific methods referenced from Duan et al. (2018). Additionally, the data were analyzed using a t-test.

#### 2.2.3 Root sampling method of plants

After being rinsed with distilled water and blotted dry using clean filter paper, the underground roots were treated with phosphate-buffered saline to remove surface soil. Roots (0.5 g) were sampled from each treatment, with three biological replicates. Following 3–4 h of incubation in liquid nitrogen, the roots were stored at −80 °C for subsequent transcriptomic and proteomic analyses.

#### 2.2.4 Total RNA extraction and data acquisition

Purification of total plant RNA relied on ethanol precipitation and the CTAB-pBIOZOL reagent as per the producer’s protocol. A NanoDrop Spectrophotometer and an Agilent 2100 Bioanalyzer (Thermo Fisher Scientific, MA, United States), respectively, were used for the identification and quantification of total RNA. Jingjie PTM BioLab Co. Inc. (Hangzhou, China) was responsible for purifying, reverse transcribing, and sequencing the obtained RNA, along with constructing the relevant library as per the manufacturer’s protocol (Illumina, San Diego, CA).

The RNA-seq root transcript library was prepared using the Illumina^®^ Stranded mRNA Kit with 1 µg of total RNA. In brief, messenger RNA isolated using oligo (dT) beads by the poly (A) selection method was fragmented. A SuperScript Double-stranded cDNA Synthesis Kit (Invitrogen, Carlsbad, CA, United States) was responsible for the synthesis of double-stranded cDNA using random hexamer primers (Illumina). The cDNA was then end-repaired, phosphorylated, and adenylated with the ‘A’ base as per the library construction protocol (Illumina). A library of 300 Pb cDNA target fragments was selected, fusion DNA polymerase (NEB) was used to select a low-range overlap of 2% (lower range 2%), and then an amplification cycle of 15 polymerase chains (PCR) was performed. Qubit 4.0 quantification was followed by the sequencing of the paired-end RNA-seq library using a NovaSeq 6000 Sequencer (2 × 150 bp read length).

fastp with default parameters was used to control the quality of the trimmed raw paired-end reads (Chen et al., 2018). Clean data were subjected to *de novo* assembly with the assistance of the Trinity (Grabherr et al., 2011). CD-HIT and TransRate were used to filter all assembled sequences to improve assembly quality, and DIAMOND was then used to search the assembled transcripts against the NCBI non-redundant (NR), COG, and Kyoto Encyclopedia of Genes and Genomes (KEGG) protein databases, thereby retrieving the functional annotations for the most similar sequences, with a cut-off E-value <1.0 × 10^−5^. The Blast2GO program helped acquire the GO annotations of the specially assembled transcripts for the description of the biological processes (BPs), molecular functions (MFs), and cellular components (CCs) (Conesa et al., 2005). Metabolic pathway analyses relied on KEGG (Kanehisa and Goto, 2000). The raw sequencing data were submitted to the NCBI BioProject database (project No. PRJNA907928 and PRJNA907870).

The expression level of each transcript was calculated using the transcripts per million method, and the expansion between the two samples was determined. RSEM served for gene abundance quantification (Li and Dewey, 2011). Differential expression analyses relied on DESeq2 or DEGseq, confirming DEGs with |log2FC| ≥ 1 and FDR ≤0.05 (DESeq2) or FDR ≤0.001 (DEGseq) as DEGs with significance. In addition, Gene Ontology (GO) and KEGG analyses, respectively, by virtue of GOATools and KOBAS, determined DEGs presenting a significant enrichment in metabolic pathways and GO terms (Bonferroni-corrected p-value ≤0.05) versus the whole-transcriptome sequencing (Xie et al., 2011).

#### 2.2.5 Quantitative real-time PCR analysis

Nine genes significantly influencing nodule nitrogen fixation were selected from the transcriptomic data and validated via quantitative real-time polymerase chain reaction (qRT-PCR). First-strand cDNA was synthesized using the FastKing RT Kit with gDNase (TIANGEN Biotech Co., LTD., Beijing, China) following the manufacturer’s protocol. Quantitative real-time PCR was performed using the SYBR Green SuperReal PreMix Plus (TIANGEN Biotech Co., LTD., Beijing, China) on an Applied Biosystems 7500 Fast Real-Time PCR System (Monad Biotech Co., Ltd., Guangzhou, China). The thermal cycling conditions were as follows: initial denaturation at 95 °C for 15 min, followed by 40 cycles of 95 °C for 10 s and 66 °C for 32 s. Melting curve analysis was conducted from 66 °C to 95 °C after the final cycle to verify primer specificity. All reactions were run in triplicate. Relative gene expression levels were calculated using the 2^−ΔΔCT^ method (Livak and Schmittgen, 2001). Primers were designed using Primer Premier 5 software (PREMIER Biosoft, United States), and their sequences are provided in Supplementary Table S1. The *RCD-1* gene was selected as an internal reference Yin et al. (2021).

#### 2.2.6 Protein extraction

Jingjie PTM BioLab (Hangzhou, China) Co. Inc. carried out the proteomic sequencing analysis on NN and CK samples. After thorough grinding under nitrogen, the root samples were placed in a phenol extraction buffer (ratio: 1:4) and subjected to ultrasonic fragmentation. The mixture of the samples and Tris underwent 10 min of centrifugation at 5,500 g and 4 °C. The supernatants, combined with 0.1 M ammonium acetate/methanol (volume ratio: 1:5), underwent precipitation. After methanol and acetone washes, protein precipitates were re-dissolved in 8 M urea. A BCA kit was used to detect protein concentration. The amount used for enzymatic hydrolysis was kept consistent for each protein. After gradual addition of trichloroacetic acid to a final concentration of 20%, the mixture was vortexed and precipitated for 2 h at 4 °C. This was followed by 5 min of centrifugation at 4,500 g and disposal of the supernatant. The pellet underwent 2–3 rounds of precipitation in precooled acetone. Following the drying of the precipitate, TEAB was added to a final concentration of 200 mM. After ultrasound dispersion, the mixture was incubated with trypsin (1:50) for overnight enzymatic hydrolysis. After the addition of DTT at 5 mM, the mixture was subjected to half an hour of incubation at 56°C, and after the addition of IAA to 11 mM, the mixture was cultured for 15 min at room temperature in the dark.

With regard to liquid chromatography, we employed a NanoElute ultra-performance liquid system to isolate the peptides after dissolution in mobile phase A (Sun et al., 2021). Mobile phase A consisted of an aqueous solution containing 0.1% formic acid and 2% acetonitrile; mobile phase B consisted of an aqueous solution containing 0.1% formic acid and 100% acetonitrile. The liquid phase gradient was as follows: 0–70 min, 5–22% B; 70–84 min, 22–32% B; 84–87 min, 32–80% B; and 87–90 min, 80% B; the flow rate was set at 300 nL/min. The isolated peptides were injected into the capillary ion source for ionization (voltage: 2.0 kV), followed by analysis using timsTOF Pro 3 mass spectrometry. High-resolution time-of-flight mass spectrometry was used to detect and analyze the parent ions of the peptides and their corresponding secondary fragments. For the secondary mass spectrum, the scanning range was 100–1,700. The parallel accumulation–serial fragmentation (PASEF) mode was used for data collection. The collection of primary spectrograms was followed by 10 rounds of the collection of secondary spectrograms, capturing 0–5 parent ions in the PASEF mode. For tandem mass spectrometry, we set the dynamic exclusion time to 30 s to prevent the parent ions from being scanned repeatedly (Cox et al., 2009).

Protein annotation included KEGG annotation and subcellular localization. Protein pathways were annotated using the KEGG databases. The identified proteins underwent BLAST comparison (blastp, *e*-value ≤ 1*e* ^−4^), with those possessing the highest scores being annotated. In addition, the KEGG online service tool KAAS was used to assist in protein annotation within the KEGG database, and the results were integrated into the KEGG pathway database using KEGG mapper. WoLF PSORT, an updated version of PSORT/PSORT II, was used to predict subcellular localization.

#### 2.2.7 PRM analysis

The extracted proteins were subjected to proteomic analysis, following the previous description. After dissolution in mobile phase A, they were isolated using the EASY-nLC 1000 UPLC system. Mobile phase A consisted of an aqueous solution containing 0.1% formic acid and 2% acetonitrile; mobile phase B consisted of an aqueous solution containing 0.1% formic acid and 90% acetonitrile. The liquid phase gradient was as follows: 0–16 min, 8–30% B; 16–22 min, 30–40% B; 22–26 min, 40–80% B; and 26–30 min, 80% B. The flow rate was 500 nL/min. The peptides isolated under an ultra-performance liquid phase system were injected into an NSI ion source for ionization (ion source voltage: 2.1 kV), and a Q Exactive Plus Mass Spectrometer was used for relevant analysis. Orbitrap, with a scanning resolution of 17,500, was used to detect and analyze the peptide parent ions and the corresponding secondary fragments. For the primary mass spectrometer, the scanning range was 382–953 m*/z*, and the scanning resolution was 70,000. A data-dependent scan program was adopted for data collection, and the HCD collision pool presented a fragmentation energy of 27. For the primary mass spectrometer, the automatic gain control (AGC) was set to 3E6, and the maximum ion implantation time (IT) was 50 ms, while for the secondary mass spectrometer, the two parameters were set to 1E5 and 220 ms, respectively, with an isolation window of 1.6 m*/z*. Regarding peptide parameters, experimenters set the protease to trypsin [Kr/P], with a maximum of 0 missed cleavage sites; the allowed peptide length was 7–25 amino acid residues, and cysteine alkylation was treated as a fixed modification. For transition parameters, the charge states of parent and fragment ions were set to 2–3 and 1, respectively, with B and Y ion types selected. Fragment ion selection ranged from the third ion to the last, and ion matching was performed with a mass error tolerance of 0.02 Da (Glen et al., 2010). In the experiment, quantitative analysis of each protein was based on more than two unique peptides. For PRM verification of certain proteins, a single unique peptide was used due to sensitivity limitations. A heavy isotope-labeled peptide was employed to normalize of the quantitative information, followed by the relative quantitative analysis (using three biological replicates) on the target proteins. Microsoft Excel 2007 and SPSS 16.00 were used to analyze the relative protein expression. Quantitative real time-PCR and PRM data analysis were analyzed using ANOVA, and the least significant difference test was used to compare treatment means at a probability level of *p* = 0.05.

## 3 Results

### 3.1 Effect of inoculation with rhizobia on nutritional quality

**FIGURE 1:**
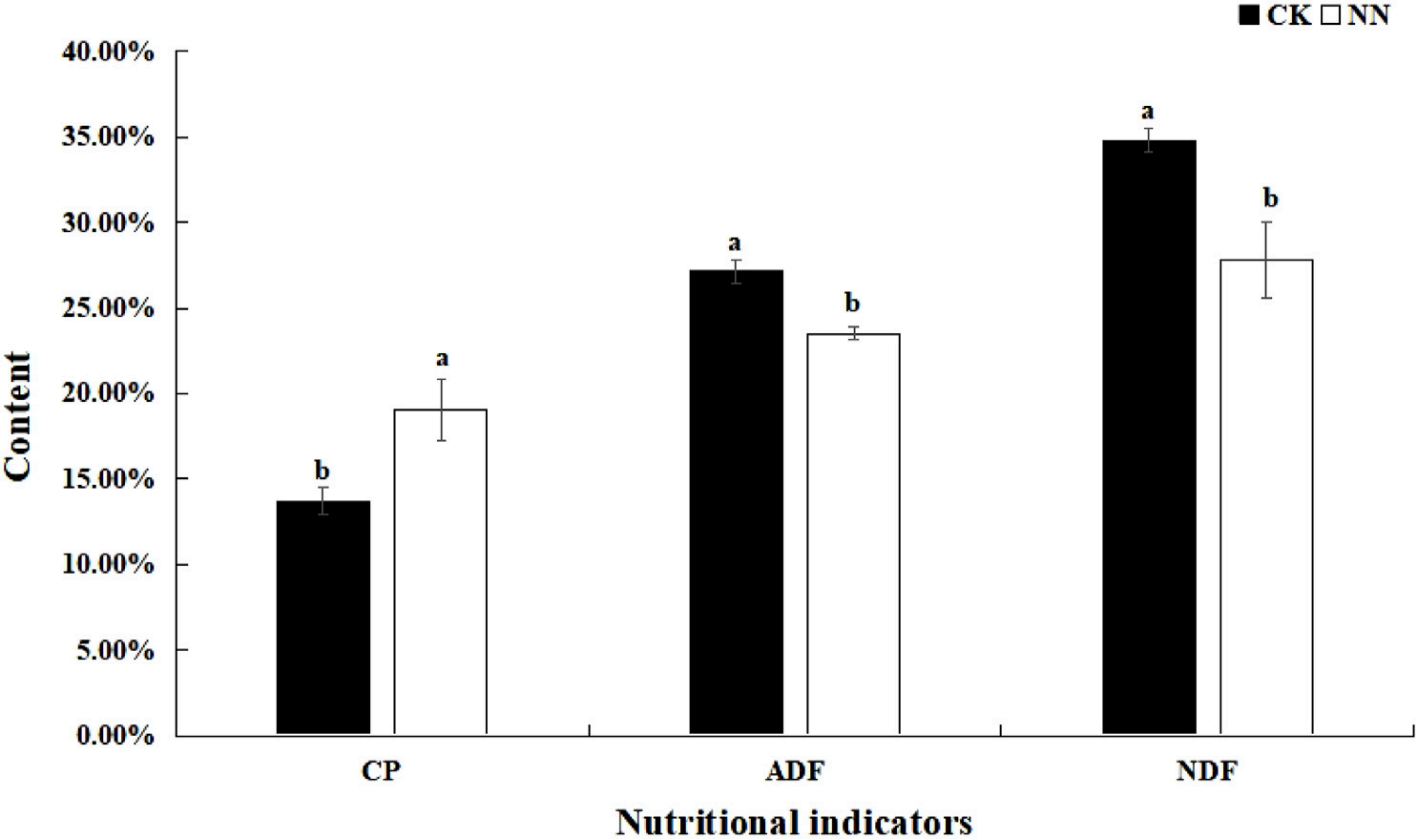
Effect of rhizobia inoculation on Caucasian clover nutritional quality. CK, control; NN, inoculation with rhizobia; CP, crude protein; NDF, neutral detergent fiber; ADF, acid detergent fiber.

Pairwise t-tests were conducted to analyze the crude protein (CP), NDF, and ADF contents of Caucasian clover. The results showed that, compared with the control group (without rhizobium inoculation; CK), the CP content of Caucasian clover in the NN treatment (inoculated with rhizobia) was significantly increased, while the NDF and ADF contents were significantly reduced. (Figure 1).

### 3.2 Transcriptomic data analysis of Caucasian clover under different treatments

The study obtained 166,528 unigenes in total, and the average length was 1,223 bp. The N50, N70, and N90 values of the unigenes were 1896, 1,300 and 586 bp, respectively (Supplementary Table S2). Supplementary Table S2 illustrates the sequence assembly after Illumina sequencing.

**FIGURE 2:**
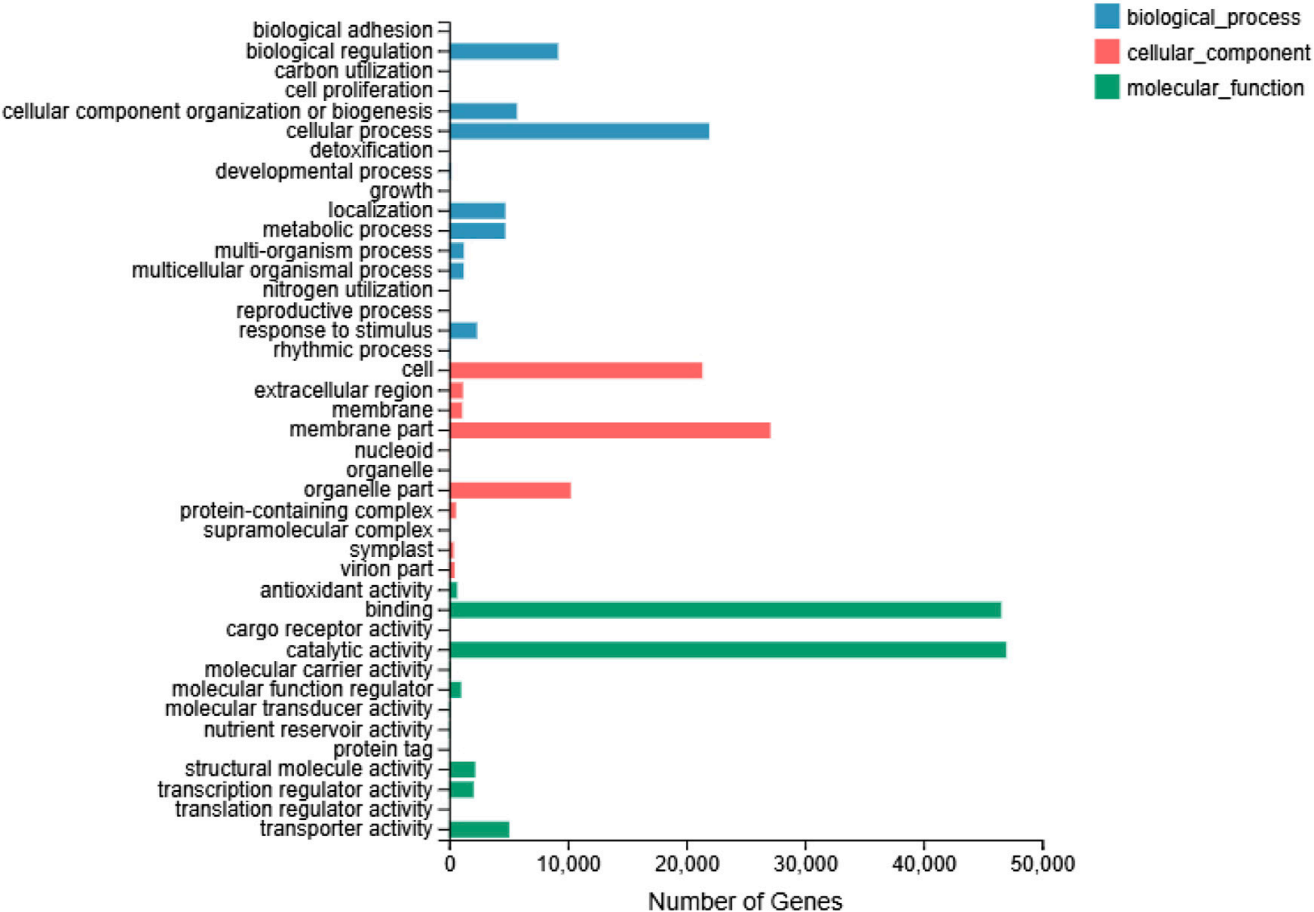
Histogram of GO classification.

In total, 89,567 genes were divided into three ontologies (Figure 2). In terms of the BP category, genes related to cellular processes (21,945), “biological regulation” (9,192), “cellular component organization or biogenesis” (5,731), and localization (4,749) were highly represented. With regard to the CC category, genes related to “membrane part” (27,103), cell (21,369), “organelle part” (10,272), and “extracellular region” (1,174) were highly represented. The highly represented GO terms (level 2) were ‘catalytic activity” (47,002), “binding” (46,570), “transporter activity” (5,075), and “structural molecule activity” (2,208) in the MF category.

In total, there were 70,280 DEGs (33,692 upregulated and 36,588 downregulated) in the roots of Caucasian clover in the NN and control groups, respectively (Supplementary Figure S1).

### 3.3 DEG functional annotation in KEGG enrichment

According to KEGG enrichment analysis on DEGs, the top five pathways were “sesquiterpenoid and triterpenoid biosynthesis” (ko00909), “steroid biosynthesis” (ko00100), “mismatch repair” (ko03430), “homologous recombination” (ko03440), and “DNA replication” (ko03030) (Table 1). They were also significantly enriched in “phenylpropanoid biosynthesis” (ko00940), “pentose and glucuronate interconversion” (ko00040), “starch and sucrose metabolism” (ko00500), and “cyanoamino acid metabolism” (ko00460) pathways.

**TABLE 1:** DEGs enriched in the KEGG pathway.

Pathway ID	Pathway name	Gene number	Rich ratio	Q value
ko03430	Mismatch repair	1,233	0.57908	9.45 × 10^−7^
ko03440	Homologous recombination	1,303	0.57636	9.45 × 10^−7^
ko03030	DNA replication	1,327	0.57046	5.33 × 10^−6^
ko00909	Sesquiterpenoid and triterpenoid biosynthesis	212	0.65094	1.77 × 10^−4^
ko03420	Nucleotide excision repair	1,439	0.55594	1.92 × 10^−4^
ko00940	Phenylpropanoid biosynthesis	1734	0.54729	6.12 × 10^−4^
ko00040	Pentose and glucuronate interconversions	1,181	0.54953	5.05 × 10^−3^
ko00100	Steroid biosynthesis	195	0.62051	6.39 × 10^−3^
ko00500	Starch and sucrose metabolism	2,228	0.53142	1.71 × 10^−2^
ko03020	RNA polymerase	1,159	0.54357	1.76 × 10^−2^
ko00460	Cyanoamino acid metabolism	969	0.54489	2.88 × 10^−2^

### 3.4 Protein profiles of Caucasian clover at different developmental stages

**FIGURE 3:**
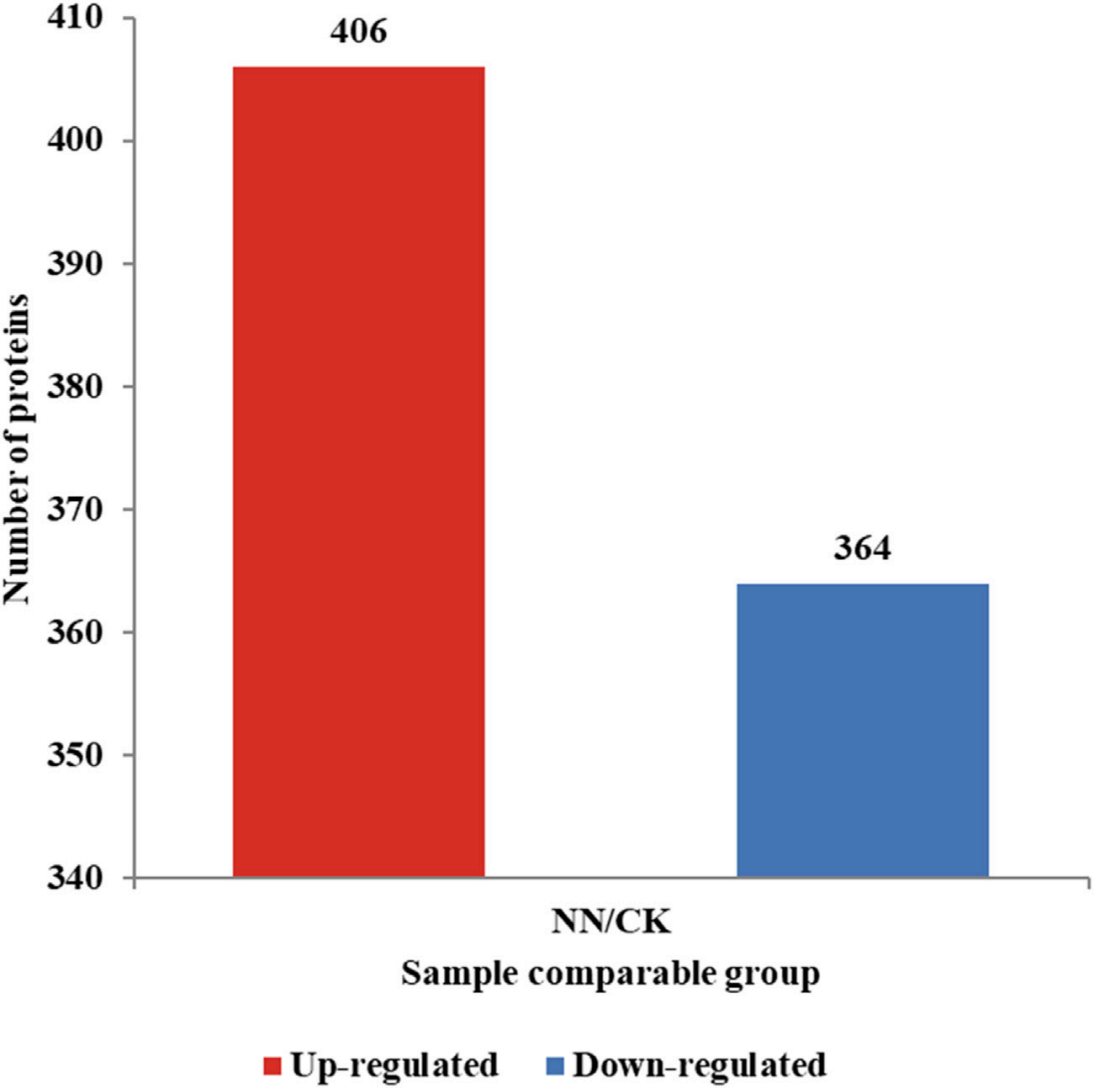
Number of DEPs in Caucasian clover roots under nodulation.

Nodular and non-nodular roots of Caucasian clover were subjected to proteomic analysis. Liquid chromatography‒mass spectrometry identified approximately 39,570 peptides, corresponding to 6,468 proteins. A 2.0-fold-change cut-off was used to determine differential expression, resulting in the identification of 770 DEPs, including 406 upregulated and 364 downregulated proteins during the comparison between nodular and non-nodular roots (Figure 3).

### 3.5 KEGG analysis of DEPs

**FIGURE 4:**
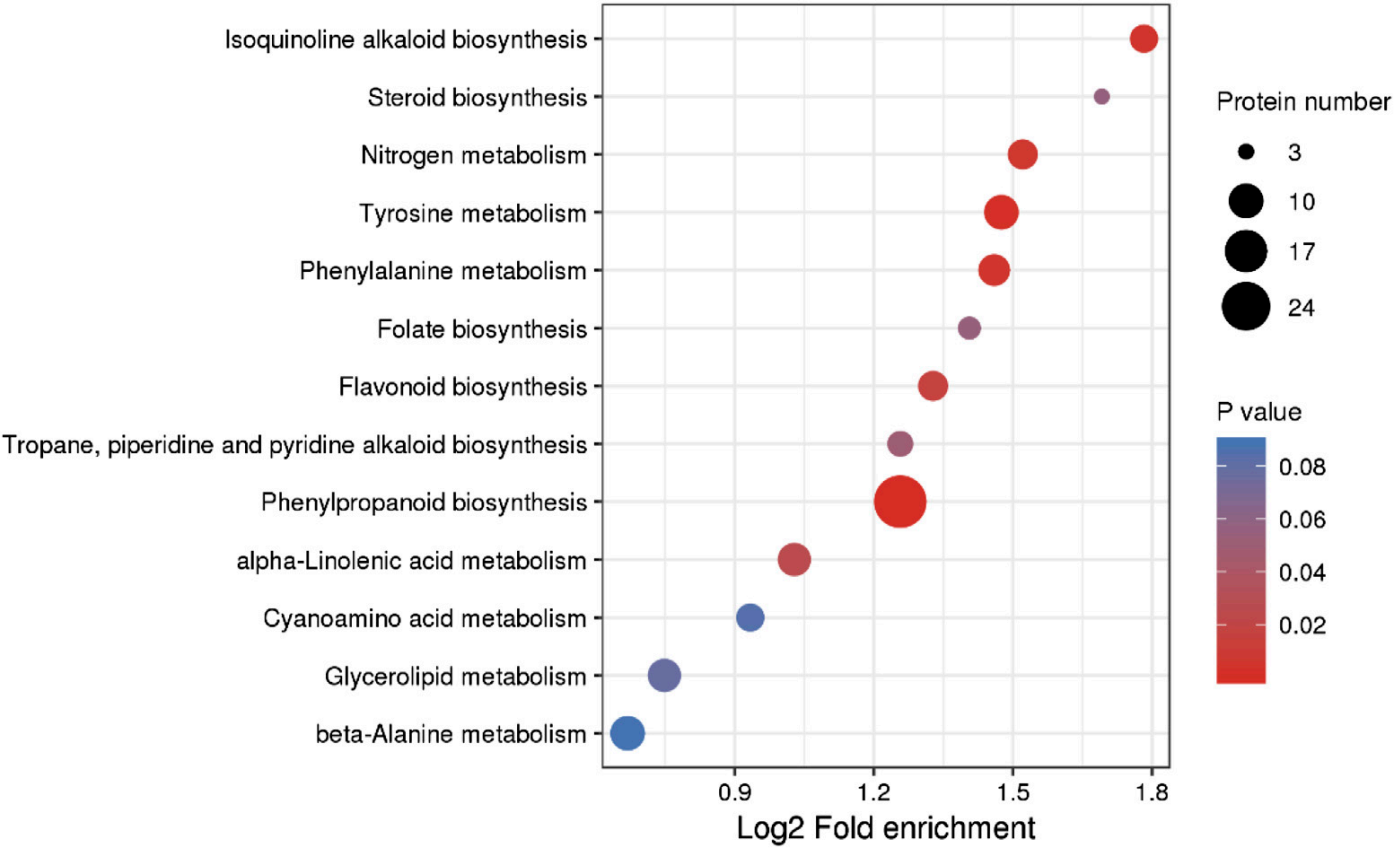
KEGG analysis of DEPs.

In total, 528 proteins were assigned to 105 pathways. The pathways associated with “phenylpropanoid biosynthesis” (ko00940), “tyrosine metabolism” (ko00350), “isoquinoline alkaloid biosynthesis” (ko00950), “phenylalanine metabolism” (ko00360), “nitrogen metabolism” (ko00910), and “flavonoid biosynthesis” (ko00941) were predominantly enriched in the NN vs. CK comparison (Figure 4).

### 3.6 Transcriptomics and proteomics crosstalk analysis

**FIGURE 5:**
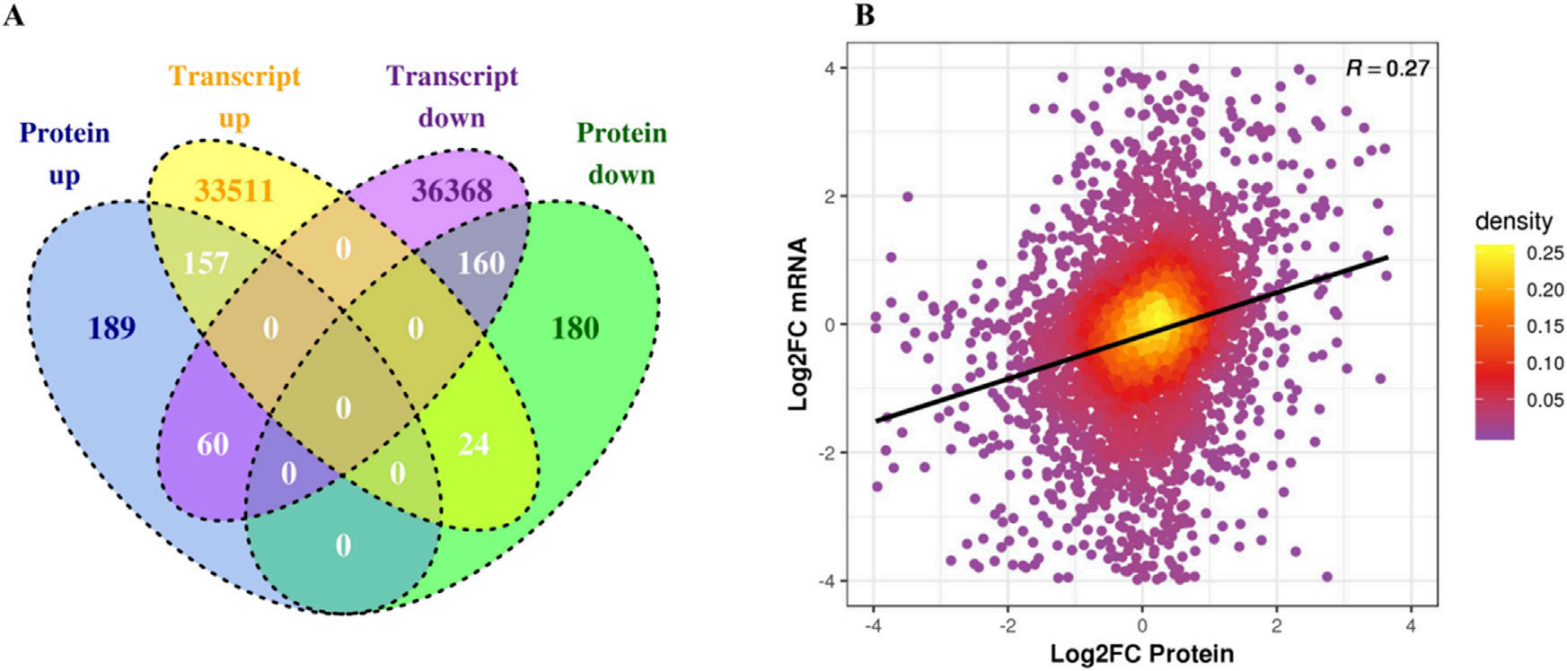
Proteomic and transcriptomic differential analysis. **(A)** Comparative analysis of differentially expressed proteins and transcripts in the comparison group Venn diagram. **(B)** Scatter plot of transcripts and their corresponding protein expressions.

In the NN vs. CK comparison, 6,468 proteins were identified in the proteomics dataset and 166,528 transcripts were identified in the transcriptomics dataset. The transcript and protein expression levels of 157 genes were upregulated, and the mRNA and protein levels of 160 genes were downregulated (Figure 5A). The expression levels of mRNA and protein show a positive correlation (Figure 5B).

**FIGURE 6:**
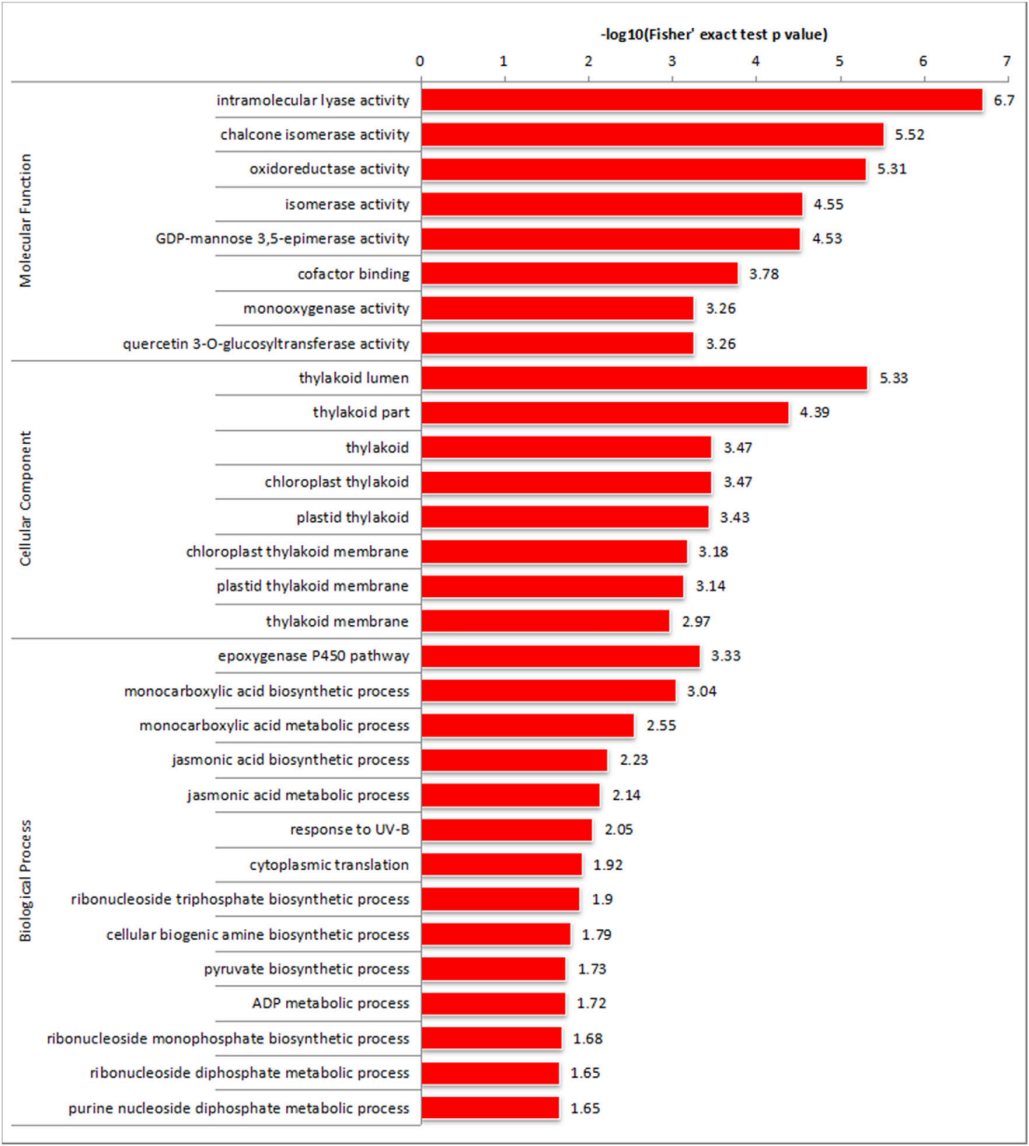
GO enrichment analysis of DEGs and DEPs from NN vs. CK.

GO enrichment analysis of upregulated DEPs revealed that in MF processes, DEP enrichment occurred in “intramolecular lyase activity” (GO:0016872), “chalcone isomerase activity” (GO:0045430), and “oxidoreductase activity” (GO:0016491); in the “CC” category, DEP enrichment could be observed in “thylakoid lumen” (GO:0031977), “thylakoid part” (GO:0044436), and “thylakoid” (GO:0009579); and in the “BP” category, the “P450 epoxygenase pathway” (GO:0019373), “monocarboxylic acid biosynthetic process” (GO:0072330), “monocarboxylic acid metabolic process” (GO:0032787), etc., presented DEG enrichment (Figure 6).

**FIGURE 7:**
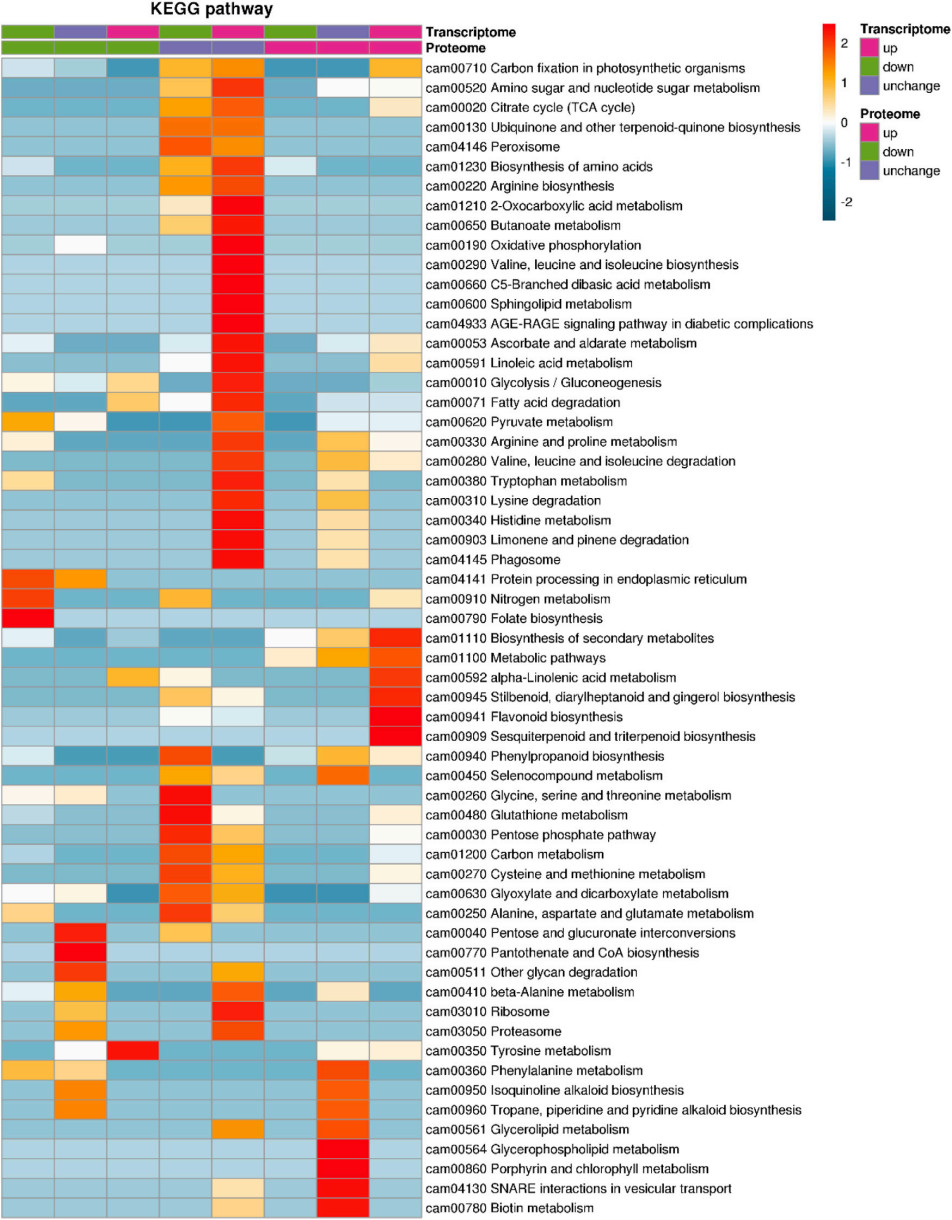
Combined analysis of transcriptomes and proteomes.

The enriched KEGG pathway for co-upregulated genes in transcriptomes and proteomics in NN vs. CK comparison were “flavonoid biosynthesis” (ko00941), “biosynthesis of secondary metabolites” (ko01110), “metabolic pathways” (ko01100), “α-linolenic acid metabolism” (ko00592), “stilbenoid, diarylheptanoid, and gingerol biosynthesis” (ko00945), and “sesquiterpenoid and triterpenoid biosynthesis” (ko00909), and the co-downregulated gene/protein-enriched pathways were “nitrogen metabolism” (ko00910), “folate biosynthesis” (ko00790), and “protein processing in endoplasmic reticulum” (ko04141) (Figure 7).

### 3.7 Expression of key pathway-related genes

**FIGURE 8:**
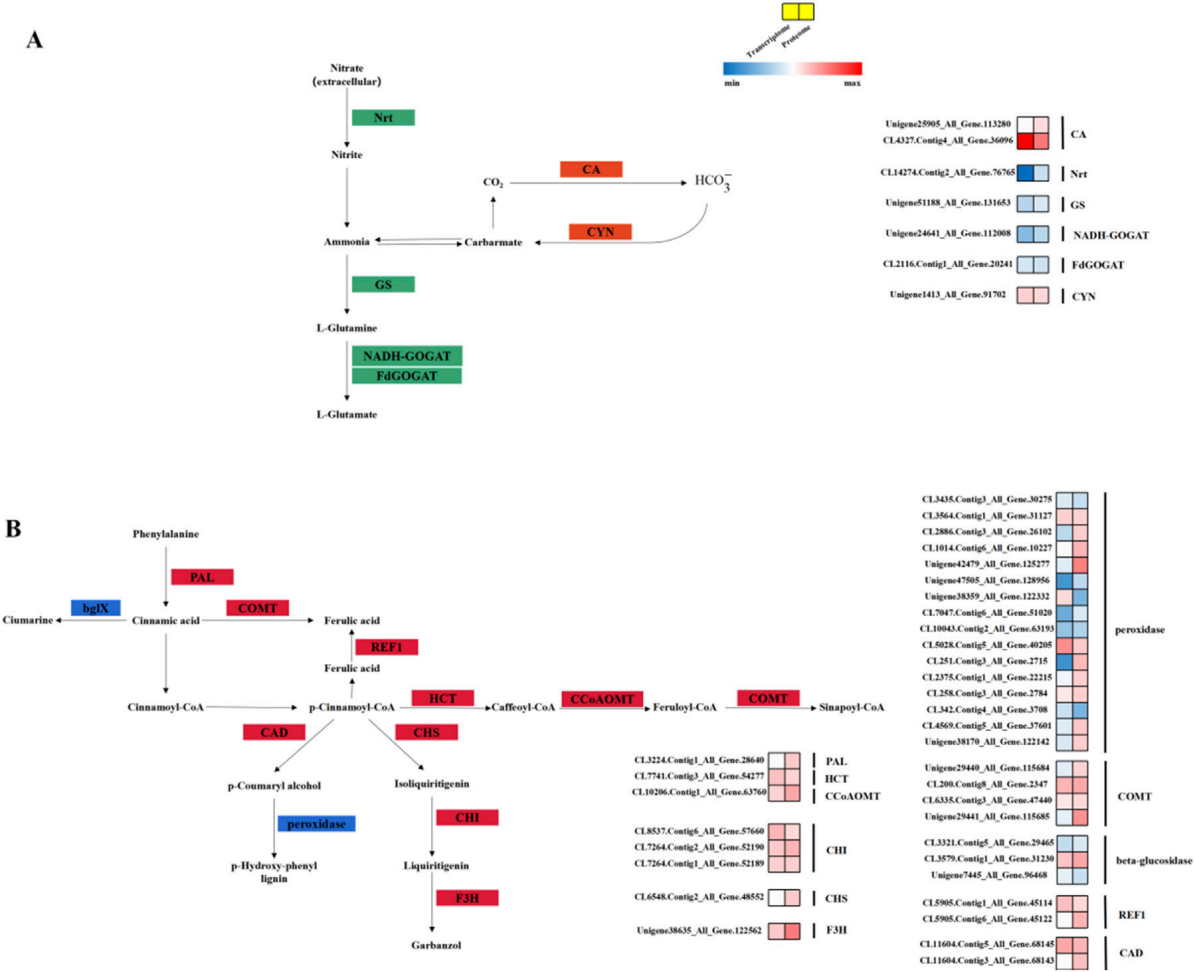
Pathways associated with nitrogen fixation in nodules of Caucasian clover. **(A)** Simplified model of nitrogen metabolic processes in Caucasian clover. **(B)** Simplified model diagram of phenylpropanoid biosynthesis and flavonoid biosynthesis in Caucasian clover. The red module expresses upregulation, the green module expresses downregulation, and the blue module indicates both up- and downregulation. Nrt, nitrate/nitrite transporter; GS, glutamine synthetase; FdGOGAT, glutamate synthase (ferredoxin); NADH-GOGAT, glutamate synthase (NADPH/NADH); CA, carbonic anhydrase; CYN, cyanate lyase; PAL, phenylalanine ammonia lyase; F3H, flavanone 3‐hydroxylase; COMT, caffeate/5‐hydroxyferulate 3‐O‐methyltransferase; CAD, cinnamyl alcohol dehydrogenase; REF, coniferyl-aldehyde dehydrogenase; HCT, hydroxycinnamoyl‐CoA shikimate/quinate hydroxycinnamoyl transferase; CCoAOMT, caffeoyl CoA 3‐O‐methyltransferase; CHS, chalcone synthase; CHI, chalcone isomerase.

To investigate the nitrogen-fixation mechanism in Caucasian clover, this study focused on genes associated with the nitrogen metabolism, phenylpropanoid metabolism, and flavonoid biosynthesis pathways. During nitrogen metabolism, the expression of DEGs encoding Nrt, GS, NADH-GOGAT, and FdGOGAT was downregulated, and the DEP content was similarly reduced; meanwhile, the expression of DEGs encoding CA and CYN presented an obvious increase and a similar upregulation in the proteome. The expression of DEGs encoding PAL, COMT, HCT, CAD, CHS, CCoAOMT, CHI, and F3H was upregulated during phenylpropanoid metabolism and flavonoid biosynthesis, with a similar upregulation observed at the proteomic level (Figure 8).

### 3.8 Protein validation by PRM

**FIGURE 9:**
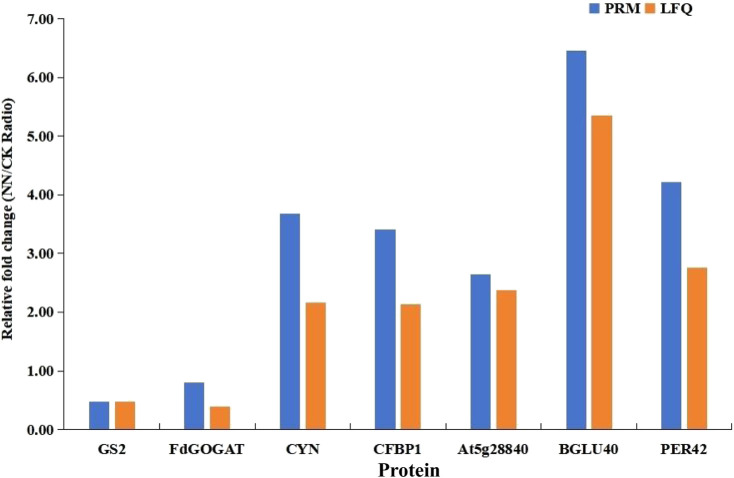
Comparison of protein expression by LFQ and PRM.

To remove possible errors, we randomly selected seven differential proteins (GS2, FdGOGAT, CYN, CFEP1, At5g28840, BGLU40, and PER42) for PRM analysis to validate the LFQ results. The results of the targeted proteins detected by PRM were in agreement with those analyzed in the test, suggesting that the proteomics sequencing results were highly reliable and reproducible (Figure 9).

### 3.9 Accuracy of qRT-PCR verification results

**FIGURE 10:**
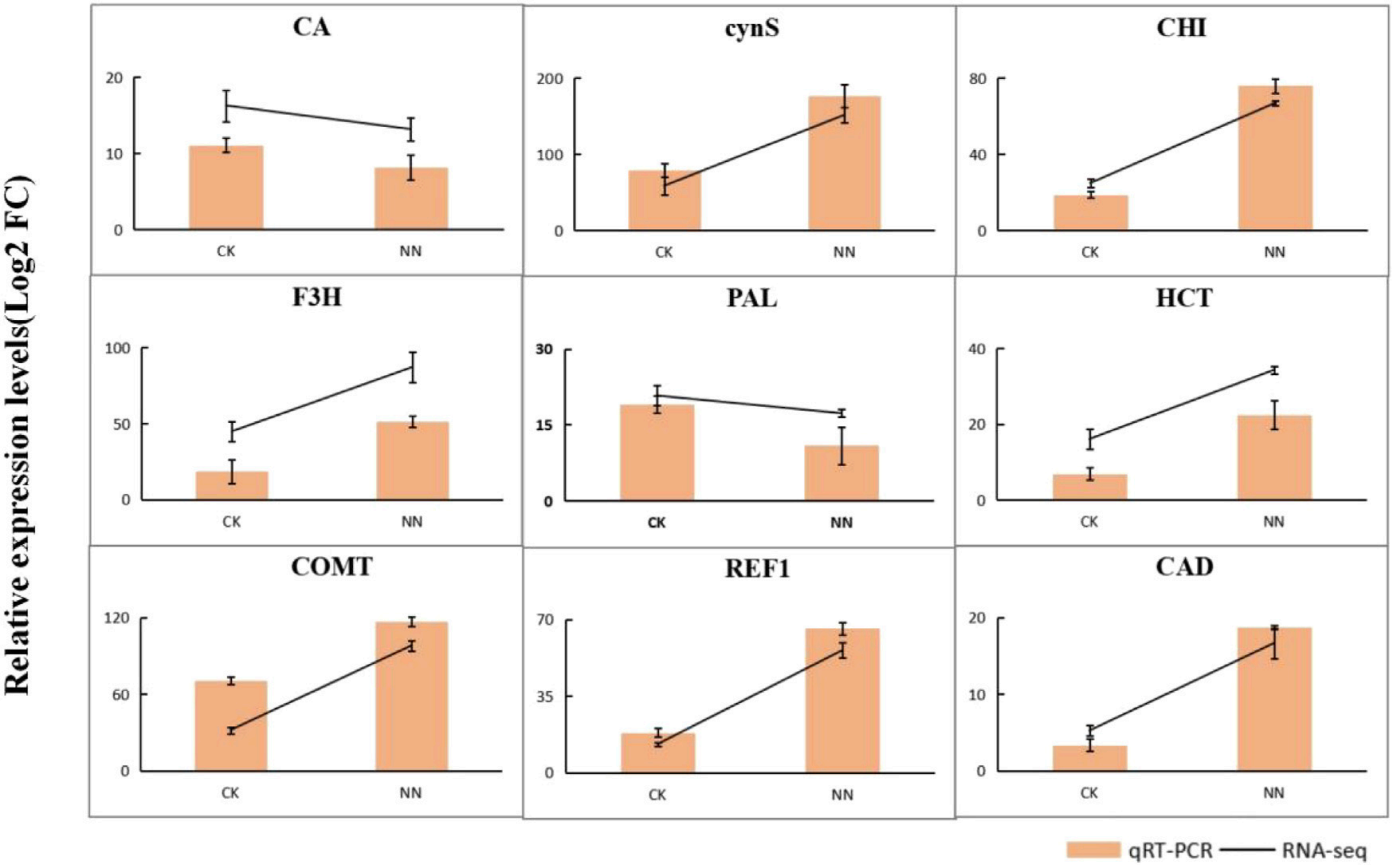
Comparison of qRT-PCR verification and FPKM value. The bar graph indicates the relative expression levels of 10 differentially expressed genes in qRT-PCR, and the folded line indicates the FPKM values of 10 differentially expressed genes in the transcriptome.

In this study, we identified genes associated with nitrogen metabolism and flavonoid biosynthesis during symbiotic nitrogen fixation in *Trifolium ambiguum* Bieb. The reliability of RNA-seq results and the DEGs selected was validated using qRT-PCR (Figure 10).

## 4 Discussion

Previous studies showed that Caucasian clover could only form a nitrogen-fixing symbiosis with certain rhizobia, a characteristic that limits its establishment (Hely, 1957; Kannenberg and Elliott, 1962). Caucasian clover is more vigorous and resistant to adversity than white clover (Watson et al., 1996), and because of its selective specificity for rhizobia, it must be inoculated with specific rhizobia in areas outside its native range to produce nodule fixation. More studies have been conducted on nodule nitrogen fixation in legumes than on Caucasian clover. Building upon this foundation, our study is the first one examining nitrogen fixation by nodulation in Caucasian clover using transcriptomic and proteomic techniques.

The nutritional quality of forage crops directly influences livestock growth, development, and the quality of animal products. Our findings revealed that rhizobial inoculation significantly increased the crude protein content and significantly decreased the NDF and ADF content in Caucasian clover. These results align with previous studies on other legumes (Li et al., 2016), indicating a high degree of compatibility between the inoculated rhizobia and *T. ambiguum*, thereby enhancing its nutritional quality.

Rhizobial inoculation induced numerous DEGs and DEPs in Caucasian clover, suggesting the involvement of specific genes and proteins in the nodulation and nitrogen fixation processes. DEG enrichment primarily occurred in sesquiterpenoid, triterpenoid, and steroid biosynthesis. Signal-regulating substances, such as flavonoids and terpenoids, are produced during the interaction between legumes and rhizobia, and terpenoids are not only involved in plant defense and growth (Mumm et al., 2008; Umehara et al., 2008; Zhou et al., 2015) but also induce *nod* genes to be expressed, promoting the interaction between legumes and rhizobacteria (Ali et al., 2021), which conforms to the findings of our study. Nitrogen fixation must occur under microaerobic conditions because oxygen generated by photosynthesis inactivates the oxygen-labile nitrogenase enzyme, hindering the process. Some rhizobial strains form specialized structures, such as bacteroid-containing vesicles, concomitant with nitrogen fixation within nodules (Fay, 1992).

Following symbiosis establishment, the host plant supplies sucrose to the rhizobia, which convert it into energy and organic acids for their metabolism. In return, the rhizobia fix atmospheric nitrogen and provide reduced nitrogen compounds to the host (Day et al., 2001). We observed a significant increase in CA activity in inoculated Caucasian clover, which is consistent with findings during symbiotic nitrogen fixation in soybean (Kavroula et al., 2000). This increase is likely due to the high demand for bicarbonate during symbiotic nitrogen fixation; elevated CA expression and activity facilitate the conversion of CO_2_ to bicarbonate, sustaining the process. Future research should focus on the mechanisms of bicarbonate supply in symbiotic nitrogen fixation.

Inorganic nitrogen in plants mainly originates from nitrate, and nitrate uptake in soil occurs through the combined action of specific nitrate transporters. Nrt is an important component of the NO_3_^−^ transport system for root uptake (Li et al., 2007). Interestingly, rhizobial inoculation downregulated Nrt expression in Caucasian clover. Previous research indicates that in *Lotus japonicus*, Nrt proteins positively regulate symbiotic nitrogen fixation at low nitrate concentrations (Valkov et al., 2020), suggesting that low levels of nitrate (NO_3_^−^) can promote nodule development and nitrogen fixation, although the underlying mechanisms remain largely unknown. Whether this downregulation results from the initiation of symbiotic nitrogen fixation requires further investigation.

Root nodule formation in legumes is a process of interaction between the host plant and rhizobia and involves the expression and regulation of several genes in a complex series of regulatory networks. The interaction between legumes and rhizobia usually begins in nitrogen-deficient soils (Redmond et al., 1986; Peters et al., 1986), where legumes secrete flavonoid metabolites and rhizobia use flavonoids as recognition signals for synthesis and release through *nod* genes (D’Haeze and Holsters, 2002). Flavonoids in legume root secretions are chemo-attractive to rhizobia under low-nitrogen conditions (Cooper, 2004; Graham, 1991). Flavonoids are a class of plant phenolic compounds present in all land plants, playing vital roles in different aspects of plant life, including UV protection, pollinator attraction, seed dispersal through pigmentation, defense, and signaling between plants and microorganisms. Flavonoid is a secondary plant metabolite synthesized by the phenylpropanoid pathway (Ferrer et al., 2008). Phenylpropanoid is a physical barrier and mechanical support for the cell wall and a major component of lignin, the accumulation of which is necessary for vascular bundle development in root nodules. Therefore, the expression of the phenylpropanoid pathway genes is responsible for vascular bundle synthesis in root nodules (Takanashi et al., 2012). In the present study, white clover inoculated with rhizobacteria showed significant increases in PAL, HCT, COMT, and CCoAOMT levels. Inoculation with rhizobacteria leads to elevated plant lignin content, which is primarily caused by the expression of PAL and HCT (Zhang et al., 2016), conforming to the findings of our study. COMT is a downstream product of the lignin biosynthesis pathway that acts on F5H, and COMT expression may increase the plant lignin content (Guo et al., 2001).

CHS and CHI catalyze the first and second steps, respectively, in flavonoid biosynthesis and are key enzymes in this pathway. During the Caucasian clover nodulation nitrogen fixation, CHI and CHS contents were significantly increased, and the rhizobial factor was a key factor in promoting CHS expression and flavonoid accumulation in legumes. *Nod*-factor-mediated signal transduction causes CHS production in plant roots, thus promoting flavonoid production (Khandual, 2007). Silencing *CHS* genes in *Medicago truncatula* reduced root flavonoid synthesis and abolished nodulation, which could be restored by exogenous flavonoid application, demonstrating the critical role of CHS in symbiotic nitrogen fixation (Wasson et al., 2006). Similarly, overexpression of the soybean *CHI* gene significantly increased nodule number and isoflavonoid content. In our study, the significant increases in CHI and CHS levels during nodulation and nitrogen fixation in Caucasian clover indicate their pivotal roles in the symbiotic process.

## 5 Conclusion

After inoculation with rhizobia in Caucasian clover, we found that DEGs and DEPs were mainly enriched in metabolic pathways such as flavonoid biosynthesis, secondary metabolite biosynthesis, and nitrogen metabolism. Among them, CA, CYN, PAL, COMT, CCoAOMT, CHS, and CHI in nitrogen metabolism, flavonoid biosynthesis, and phenylpropanoid biosynthesis pathways may serve as key factors affecting nodulation and nitrogen fixation in *Trifolium ambiguum*. Follow-up studies can continue to conduct detailed analyses of the genes and proteins identified in this research.

## Data Availability

The datasets presented in this study can be found in online repositories. The names of the repository/repositories and accession number(s) can be found at: https://www.ncbi.nlm.nih.gov/, PRJNA907928.
