# Supplementary material for: Mechanism of nodulation and nitrogen fixation in Caucasian clover (Trifolium ambiguum Bieb.) based on transcriptomics and proteomics analyses
Source: Front Genet. 2025 Jul 24;16:1600377. doi: 10.3389/fgene.2025.1600377 (PMC12328194; doi:10.3389/fgene.2025.1600377)
Supplement: Supplementary file 1 [file Table1.docx]

**Table S1 qRT-PCR primer sequences**

| **Gene** | **Gene ID** | **Forward primer sequence（5'→3‘）** | **Reverse primer sequence（5'→3’）** |
| --- | --- | --- | --- |
| CA | Unigene25905_All_Gene.113280 | CCACTCCTTCTCACTGTGTCACTC | TCTTTACCTAGCTTTGGCACTTCA |
| cynS | Unigene1413_All_Gene.91702 | GGAAGGGTGTCATAGTTAGTGGC | GCAGAAGGTGTTGGTTTTGTTGT |
| CHI | CL8537.Contig6_All_Gene.57660 | CGGACGAATGAACAATGACAAC | GGCGGGAAAAGCTTAGAAGAAT |
| F3H | Unigene38635_All_Gene.122562 | GGCAGCTCCATTTCCTTCTTATC | CCAGGGGGACTTACCTATTTTTC |
| PAL | CL3224.Contig1_All_Gene.28640 | CAACACAAATCCTCCTTCCAGC | GTTTACATCCATCCACACCCCT |
| HCT | CL7741.Contig3_All_Gene.54277 | ATAACCCCAGTTCCAATCCACTA | TAGTTCCATATTTAGACCCACCACC |
| COMT | Unigene29440_All_Gene.115684 | TGTTGTGCTCCTTGTGTTCCTC | TGACATTCCGATTGTTTCTGGT |
| REF1 | CL5905.Contig1_All_Gene.45114 | GTATGGCTGAAGCAAGGAAAGAAG | CAAGGTCAATAGAGATAAGACCGG |
| CAD | CL11604.Contig5_All_Gene.68145 | TATAACCAACCCTTAGCCACTCTT | CTTCTCCTCTACCTTTACATCCGA |

**Table S2. Summary of sequence assembly after** I**llumina sequencing**.

| **Sample** | **Raw Reads (M)** | **Clean Reads (M)** | **Clean Bases(Gb)** | **Q20(%)** | **Q30(%)** | **Clean Reads Ratio(%)** | **GC(%)** |
| --- | --- | --- | --- | --- | --- | --- | --- |
| CK_1 | 49.08 | 43.83 | 6.57 | 97.33 | 90.17 | 89.3 | 38.12 |
| CK_2 | 49.08 | 44.5 | 6.67 | 97.24 | 89.89 | 90.67 | 38.1 |
| CK_3 | 49.08 | 44.19 | 6.63 | 97.22 | 89.91 | 90.04 | 38.08 |
| NN_1 | 49.08 | 43.65 | 6.55 | 97.37 | 90.31 | 88.94 | 38.83 |
| NN_2 | 52.59 | 46.51 | 6.98 | 97.3 | 90.07 | 88.45 | 38.6 |
| NN_3 | 49.08 | 43.47 | 6.52 | 97.31 | 90.14 | 88.57 | 38.6 |

**Table S3. Length distribution of the unigene**.

| **Sequence Size(nt)** | **Unigene Number** |
| --- | --- |
| 200~500 | 53806 |
| 500~1000 | 35260 |
| 1000~2000 | 45616 |
| 2000~3000 | 20444 |
| ≥3000 | 11402 |
| Total | 166528 |
| Mean Length | 1223 |
| Total Length | 203784200 |
| N50 | 1896 |
| N70 | 1300 |
| N90 | 586 |
