# Supplementary material for: Mechanism of nodulation and nitrogen fixation in Caucasian clover (Trifolium ambiguum Bieb.) based on transcriptomics and proteomics analyses
Source: Front Genet. 2025 Jul 24;16:1600377. doi: 10.3389/fgene.2025.1600377 (PMC12328194; doi:10.3389/fgene.2025.1600377)
Supplement: Supplementary file 2 [file DataSheet1.docx]

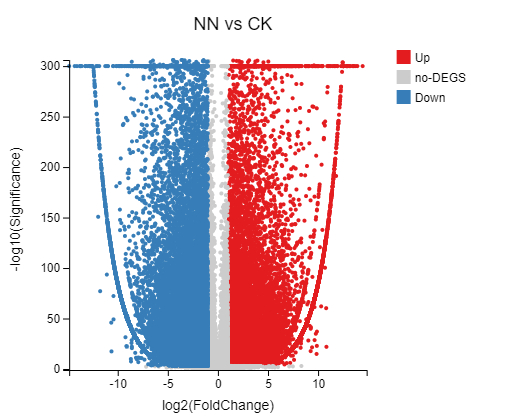


**Figure S1 Volcano plot of differentially expressed genes in nodule plants after RNA-seq analysis**


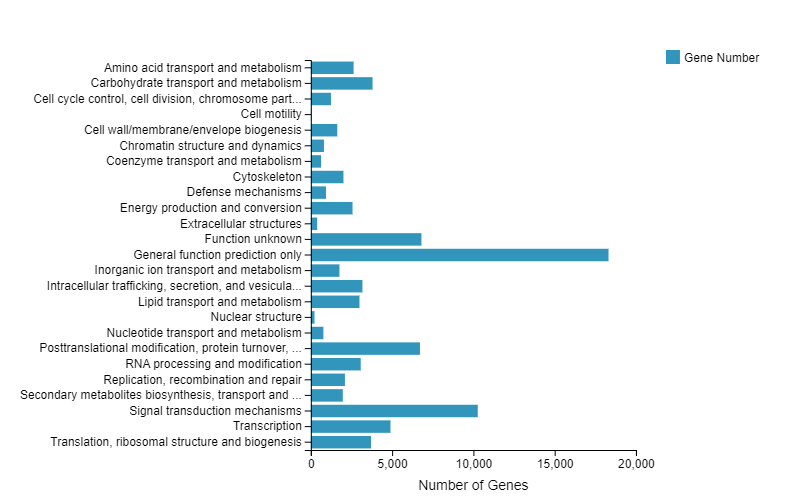


**Figure S2** **Eukaryotic orthologous groups (KOG) annotation**.
